# Characterization of surface-exposed structural loops as insertion sites for foreign antigen delivery in calicivirus-derived VLP platform

**DOI:** 10.3389/fmicb.2023.1111947

**Published:** 2023-02-27

**Authors:** Mirosława Panasiuk, Milena Chraniuk, Karolina Zimmer, Lilit Hovhannisyan, Vasil Krapchev, Grażyna Peszyńska-Sularz, Magdalena Narajczyk, Jan Węsławski, Agnieszka Konopacka, Beata Gromadzka

**Affiliations:** ^1^Department of In Vitro Studies, Institute of Biotechnology and Molecular Medicine, Gdańsk, Poland; ^2^Nano Expo Sp z.o.o, Gdańsk, Poland; ^3^Intercollegiate Faculty of Biotechnology, University of Gdańsk and Medical University of Gdańsk, Gdańsk, Poland; ^4^Faculty of Health Sciences, Department of Biochemistry and Molecular Biology, University of Bielsko-Biala, Bielsko-Biala, Poland; ^5^Tri-City Central Animal Laboratory Research and Service Center, Medical University of Gdańsk, Gdańsk, Poland; ^6^Laboratory of Electron Microscopy, Faculty of Biology, University of Gdańsk, Gdańsk, Poland; ^7^Laboratory of Molecular and Cellular Neurobiology, International Institute of Molecular and Cell Biology, Warsaw, Poland; ^8^Department of Pharmaceutical Microbiology, Faculty of Pharmacy, Medical University of Gdańsk, Gdańsk, Poland

**Keywords:** vaccine development, VLP platform, chimeric virus-like particles, foreign antigen, influenza A virus, calicivirus, norovirus, immunogenicity

## Abstract

Chimeric virus-like particles (cVLPs) show great potential in improving public health as they are safe and effective vaccine candidates. The capsid protein of caliciviruses has been described previously as a self-assembling, highly immunogenic delivery platform. The ability to significantly induce cellular and humoral immunity can be used to boost the immune response to low immunogenic foreign antigens displayed on the surface of VLPs. Capsid proteins of caliciviruses despite sequence differences share similar architecture with structural loops that can be genetically modified to present foreign epitopes on the surface of cVLPs. Here, based on the VP1 protein of norovirus (NoV), we investigated the impact of the localization of the epitope in different structural loops of the P domain on the immunogenicity of the presented epitope. In this study, three distinct loops of NoV VP1 protein were genetically modified to present a multivalent influenza virus epitope consisting of a tandem repeat of M2/NP epitopes. cVLPs presenting influenza virus-conserved epitopes in different localizations were produced in the insect cells and used to immunize BALB/c mice. Specific reaction to influenza epitopes was compared in sera from vaccinated mice to determine whether the localization of the foreign epitope has an impact on the immunogenicity.

## 1. Introduction

In recent years, vaccine development has advanced significantly, with the focus shifting from the use of whole microorganisms to safer subunit vaccines containing only their antigenic proteins. Despite their safety and effectiveness, there are several key disadvantages to the use of subunit vaccinations. Due to misfolding of the targeted antigen or inadequate immune system presentation, subunit vaccines are often less immunogenic than whole pathogens and require additional use of adjuvants to establish both innate and adaptive immunity (Moyle and Toth, 2013; Karch and Burkhard, 2016; Brisse et al., 2020; Pollard and Bijker, 2021). There has been a great deal of research into alternative approaches that address those issues. Among others, nanoscale virus-like particles (VLPs), which showed several therapeutic, immunogenic, and diagnostic uses, are regarded as the most promising platform for vaccine development among subunit vaccines (Frietze et al., 2016; Ong et al., 2017; Castilho et al., 2022).

Virus-like particles are biological nanostructures that maintain the same antigenic conformation as the original virus but lack genetic material and thus are not infectious (Roldão et al., 2010). VLPs are highly immunogenic because they represent the pathogen-associated molecular patterns (PAMPs) that target specific immune cells and thus can stimulate immune responses and enhance the delivery of antigens, acting as “self-adjuvant” (Braunstein et al., 2018; Nie et al., 2018). One of the main uses of VLPs is to provide a delivery system for various compounds such as DNA, siRNA, protein/peptide, and drugs. It is widely accepted that the delivery of foreign DNA by VLPs is a very useful method for vaccination and gene therapy (Shirbaghaee and Bolhassani, 2016; He et al., 2022). VLPs possess outstanding physical properties as nanocarriers such as controllable self-assembly/disassembly allowing for efficient cargo packaging. Also, their interior surface can be easily modified through either chemical modification or genetic reengineering to improve the cargo encapsulation process and allow tracing of the nanoparticle intake into the targeted cells (Rohovie et al., 2017; Schwarz et al., 2017; Mejía-Méndez et al., 2022).

In addition, due to their structural flexibility, VLPs can be used as a platform for the integration of foreign antigens at the exterior of the nanostructure through genetic engineering or by chemical coupling/conjugation resulting in multivalent chimeric VLPs (cVLPs; Lei et al., 2020; Watanabe et al., 2020). cVLPs, in addition to being used to induce immune responses against the particle itself, have been successfully used as a platform for inducing strong immune responses against inserted foreign immunogenic epitopes (Parra et al., 2012; Tan and Jiang, 2012; Pattinson et al., 2020). cVLPs carrying antigenic epitopes on their surface show great potential in vaccine development as a vaccine platform that can theoretically induce robust antibody responses targeting any organism (Zamora-Ceballos et al., 2022).

*Caliciviruses* were studied extensively for their ability to self-assemble into VLPs that were morphologically and antigenically identical to the infectious particles. All viruses of this family are structurally similar with capsid that contains 180 monomers of the major structural capsid protein, organized into 90 dimeric capsomers arranged into T = 3 icosahedral structure (Prasad et al., 1994; Thouvenin et al., 1997; Chen et al., 2004).

In this study, using VLPs assembled from 180 copies of major capsid protein VP1 of a norovirus (NoV), a member of the *Calicivirus* family, we examined whether the localization of the insertion site of the foreign antigens has an impact on the immunogenicity of the presented epitope. Previous reports showed that small (few amino acids long) and large (over 250 amino acids long) antigens can be successfully incorporated through genetic engineering into structural loops of NoV P particles which are 24-mer subviral particles with T = 1 icosahedral symmetry, composed by 24 P domains of NoV capsid protein (Xia et al., 2011; Tan et al., 2011a,b; Tan and Jiang, 2019; Schneider et al., 2022). Each P domain of NoV VP1 has three distinct structural loops: loop 1 (I293-H297), loop 2 (T371-D374), and loop 3 (D391-N394), which have been shown useful for antigen presentation. Other members of *Caliciviridae,* such as RHDV or FCV, share similar capsid structures with equivalent surface-exposed loops (Bárcena et al., 2004; Luque et al., 2012; Moreno et al., 2016; Zamora-Ceballos et al., 2022). Here, viral-sized NoV VLPs with T = 3 icosahedral symmetry, composed of 180 copies of the NoV capsid protein, were used to explore the potential of NoV cVLPs as a vaccine candidate, as well as to investigate whether the immune response to the antigen is affected by the epitope’s spatial presentation and availability. For this study, 110 amino acid-long multivalent influenza virus epitope, consisting of tandem repeats of M2/NP epitopes (Wang et al., 2019; Mytle et al., 2021), was inserted into each of the three structural loops of NoV VP1 resulting in three chimeric VLPs with surface-exposed epitopes.

We have demonstrated that the localization of the presented epitope can at some level impact its presentation and immunogenicity. Our findings can provide insight to effectively enhance vaccine development.

## 2. Materials and methods

### 2.1. Cell lines

Sf9 (*Spodoptera frugiperda,* ATCC: CRL-1711) insect cells were cultured in serum-free Insect SFX media (Cytiva, Marlborough, USA) supplemented with an antimycotic-antibiotic cocktail (Thermo Fisher Scientific, Waltham, USA). The suspension culture was maintained in a humidified incubator with shaking at 27°C.

### 2.2. Genetic constructs

Gene sequence of full-length NoV VP1 capsid protein (the GII.4 NoV 2012 pandemic variant Hu/GII.4/Sydney/NSW0514/2012/AU) was modified by inserting influenza virus multivalent M2/NP epitope SLLTEVETPIRNEWGCR CNDSSDSLLTEVETPIRNEWGCRCN GSSDSLLTEVETPTRSEWEC RCSDSSDSLLTEVETPTRNEWECRC SDSSDASNENIETML PFEKS TVM between amino acids 293–297 (loop 1), 371–374 (loop 2), or 391–394 (loop 3) or into all three loops (positive control). The codon-optimized (for insect expression) genes encoding for NoV-M2/NP recombinant proteins and empty NoV platform were synthesized (GeneArt, Thermo Fisher Scientific, Waltham, USA). Synthetic genes were cloned into the EcoRI and BamHI restriction enzyme recognition sites present in the multiple cloning site downstream of the polyhedrin promoter of the pFastBac1 expression vector (Invitrogen).

Recombinant NoV-M2/NP bacmids were generated using a Bac-to-Bac baculovirus expression system (Invitrogen, Waltham, USA). *E. coli* DH10-Bac competent cells were transformed with recombinant pFastBac1 vectors to generate recombinant bacmid DNA. Homologous recombination of the gene of interest into the bacmid was confirmed by PCR according to the manufacturer’s protocol. Recombinant bacmids were purified and transfected into Sf9 cells. After 6 days, recombinant baculoviruses were harvested, amplified, and titrated.

### 2.3. SDS-PAGE and Western blotting

Culture media from NoV-M2/NP baculovirus-infected Sf9 insect cells was harvested 96 h post-infection. Culture media from cells infected with an empty NoV platform was used as a control. Samples were loaded on 10%–20% precast WedgeWell Gel (Thermo Fisher Scientific, Waltham, USA) and run at the constant voltage of 165 V. After electrophoresis, semi-dry electrotransfer of proteins onto nitrocellulose membranes was performed using Trans-Blot Turbo RTA Mini 0.2 μm Nitrocellulose Transfer Kit (Bio-Rad, Hercules, USA). Membranes were then blocked for 1 h in a 5% semi-skimmed milk solution (5% milk/TBS/0.01% Tween 20) and incubated overnight with primary antibodies solution: rabbit polyclonal anti-NoV serum (obtained by vaccination of rabbit with insect cell-derived NoV VP1), mouse monoclonal anti-M2 (Santa Cruz Biotechnologies, Dallas, USA) rabbit anti-NP polyclonal antibody (Genetex, Irvine, USA), or anti-β-actin antibodies (Abcam, Waltham, USA) in 5% milk/TBS/0.01% Tween 20. Then, the membranes were washed (TBS/0.01% Tween 20) and incubated with a solution of AffiniPure goat anti-rabbit or anti-mouse antibodies (Jackson Immuno Research, Ely, United Kingdom) in 5% milk/TBS/0.01% Tween 20. The reaction was developed with SuperSignal West Pico PLUS Chemiluminescent Substrate (Thermo Fisher Scientific, Waltham, USA).

### 2.4. Protein purification

The Sf9 insect cells were infected with recombinant NoV-M2/NP baculovirus at MOI = 5 and cultured for 96 h. Culture media was harvested and concentrated using Amicon 100 k Centrifugal Filter Units (Merck Millipore, Burlington, USA). Culture media from cells infected with an empty NoV platform was used as a control. Concentrated samples were layered on OptiPrep gradient (Sigma-Aldrich, St. Louis, USA) formed in the ultra-clear tube (2 mL of 50% (v/v) OptiPrep, 2 mL of 44% (v/v) OptiPrep, 1.5 mL of 38% (v/v) OptiPrep, 1.5 mL of 32% (v/v) OptiPrep, and 1.5 mL of 26% (v/v) OptiPrep in ultra-clear water) and ultracentrifuged at 27,000 rpm for 16 h at 4°C (SW28 Ti Swinging-Bucket Rotor, Beckman, Indianapolis, USA). Then, 500 μL fractions were collected and analyzed by Western blotting. The purity of the fractions was evaluated by SDS-PAGE with InstantBlue (Expedeon, San Diego, USA) Coomassie-based staining. Fractions containing purified protein were pulled and concentrated using Amicon 100 k Centrifugal Filter Units (Merck Millipore, Burlington, USA).

### 2.5. Transmission electron microscopy (TEM)

Purified and concentrated chimeric NoV-M2/NP VLPs were diluted in TM buffer (50 mM Tris HCl, pH 7.4, 10 mM MgCl2). To visualize, the cVLPs were absorbed on the carbon-coated grids followed by negative staining with 2% uranyl acetate. The particles were observed at 120 kV in the Tecnai Spirit BioTWIN (FEI, United States).

### 2.6. Animal immunization

A group of six BALB/c male mice (6 weeks old) were immunized subcutaneously with 15 μg (day 0) or 10 μg (days: 14, 28, and 42) of chimeric NoV-M2/NP VLPs (loop 1, loop 2, and loop 3 constructs) mixed in the 1:1 ratio with adjuvant (AddaVax, InvivoGen, San Diego, USA). The mice serving as negative controls were immunized with a 1:1 PBS–adjuvant mixture. In addition, a group of six BALB/c male mice (6 weeks old) were immunized subcutaneously with 15 μg (day 0) or 10 μg (days: 14, 28) of NoV-empty platform VLP mixed in the 1:1 ratio with adjuvant (AddaVax, InvivoGen, San Diego, USA). All experiments on animals were conducted by an accredited company (Tri-City Central Animal Laboratory Research and Service Center, Medical University of Gdańsk) in accordance with the current guidelines for animal experimentation. The protocols were approved by the Committee on the Ethics of Animal Experiments of the Medical University of Gdańsk (Permit Number: 45/2015). All surgery procedures were performed under isoflurane anesthesia, and all efforts were taken to minimize animal suffering. Sera from immunized mice were collected and pooled on day 56 after immunization.

### 2.7. Analysis of immune response to immunization with chimeric NoV-M2/NP VLPs using ELISA assay

A 96-well ELISA plate (High Binding, Clear, Sarstedt, Nümbrecht, Germany) was coated with 100 μL/well of M2 antigen-presenting VLPs (NoV VLP with M2/NP epitope in all three loops). The coated plate was incubated with shaking for 2 h at room temperature. Then, the plate was washed 4 × 5 min with 200 μL/well of wash buffer (Tris-buffered saline pH 7.2/0.1%BSA/0.05% Tween 20). Next, 100 μL/well of sera from NoV-M2/NP VLP vaccinated mice (1:100 in wash buffer) was added, and the plate was incubated for 1 h at room temperature and then washed as previously. Sera from mice vaccinated with empty NoV platform and sera from mice vaccinated with PBS served as a background control. Monoclonal anti-M2 antibody (Santa Cruz Biotechnologies, Dallas, USA) served as a positive control. Then, 100 μL/well of peroxidase-conjugated AffiniPure goat anti-mouse antibodies (Jackson Immuno Research, Ely, United Kingdom; 1:2,000 in wash buffer) were added and incubated for 1 h at room temperature. The plate was washed as previously, and 100 μL/well of HRP substrate solution was added (1-Step Turbo TMB-ELISA, Thermo Scientific, Waltham, USA). The plate was incubated in the dark until the blue color developed, and the reaction was stopped by adding 50 μL of 0.5 M sulfuric acid to each well. Signal intensity was measured at 450 nm using a plate reader (Epoch, BioTek, Winooski, USA).

### 2.8. End-point titration of different NoV-M2/NP mouse sera by ELISA assay

A 96-well ELISA plate (High Binding, Clear, Sarstedt, Nümbrecht, Germany) was coated with 100 μL/well of M2 antigen-presenting VLPs (NoV VLP with M2/NP epitope in all three loops). The coated plate was incubated overnight at 4°C. Next, the plate was washed 4 × 5 min with 200 μL/well of washing buffer (PBS/0.05% Tween 20) and blocked for 2 h with 250 μL/well of blocking buffer (3% BSA/PBS/0.05% Tween 20) at 37°C. The plate was washed as previously, and serial dilutions of pooled mouse sera (in 3% BSA/PBS/0.05% Tween 20) were added to the wells and incubated for 1 h at room temperature. Serial dilutions of sera from mice vaccinated with NoV-empty platform (1:100 in 3% BSA/PBS/0.05% Tween 20) served as a positive control. After incubation, the plate was washed as previously, and peroxidase-conjugated AffiniPure goat anti-mouse antibodies (Jackson Immuno Research, Ely, United Kingdom; in 3% BSA/PBS/0.05% Tween 20) were used for detection. Finally, following the last plate-washing step (6 × 5 min with 200 μL/well), 100 μL/well of HRP substrate solution was added (1-Step Turbo TMB-ELISA, Thermo Scientific, Waltham, USA), the plate was incubated in the dark until the blue color developed, and the reaction was stopped by adding 50 μL of 0.5 M sulfuric acid to each well. Signal intensity at 450 nm was measured using a plate reader (Epoch, BioTek, Winooski, USA).

### 2.9. Analysis of the impact of the loop localization on epitope presentation by peptide ELISA assay

A 96-well ELISA plate (High Binding, Clear, Sarstedt) was coated with 100 μL/well of M2 (SLLTEVETPIRNEWGCRCNDSSD) or NP (ASNENIETMLPFEKSTVM) peptide (synthesized by JPT Peptide Technologies, Berlin, Germany) adjusted to 20 μg/mL. The coated plate was incubated overnight at 4°C. Next, the plate was washed 4 × 5 min with 200 μL/well of washing buffer (PBS/0.05% Tween 20) and blocked for 2 h with 250 μL/well of blocking buffer (3% BSA/PBS/0.05% Tween 20) at 37°C. The plate was washed as previously, and 100 μL/well of pooled mouse sera (1:100 in 3% BSA/PBS/0.05% Tween 20) were added to the wells and incubated overnight at 4°C. Sera from mice vaccinated with NoV-empty platform and unvaccinated mice (in 3% BSA/PBS/0.05% Tween 20) served as a negative control. Monoclonal anti-M2 and rabbit anti-NP polyclonal antibody (Santa Cruz Biotechnologies, Dallas, USA) served as a positive control. After incubation, the plate was washed as previously, and peroxidase-conjugated AffiniPure goat anti-mouse antibodies (Jackson Immuno Research; in 3% BSA/PBS/0.05% Tween 20) were used for detection. Finally, following the last plate-washing step (6 × 5 min with 200 μL/well), 100 μL/well of HRP substrate solution was added (1-Step Turbo TMB-ELISA, Thermo Scientific), the plate was incubated in the dark until the blue color developed, and the reaction was stopped by adding 50 μL of 0.5 M sulfuric acid to each well. Signal intensity at 450 nm was measured using a plate reader (Epoch, BioTek, Winooski, USA).

### 2.10. Characterization of the immune response toward universal influenza epitopes recognition by ELISA assay

A 96-well ELISA plate (High Binding, Clear, Sarstedt, Nümbrecht, Germany) was coated with 100 μL/well-inactivated virus for influenza A virus of human H1N1 (VR-1736, ATCC), avian H5N2 (A/Ost/Den/72420/96, Animal Health and Veterinary Laboratories, Weybridge, United Kingdom), and avian H7N1 (A/Afri.Star./Eng-Q/938/79, Department of Poultry Diseases, National Veterinary Research Institute, Pulawy, Poland) strains. All viruses used were propagated and inactivated using 1% Triton X-100. The coated plate was incubated overnight at 4°C. Next, the plate was washed 4 × 5 min with 200 μL/well of washing buffer (PBS/0.05% Tween 20) and blocked for 2 h with 250 μL/well of blocking buffer (3% BSA/PBS/0.05% Tween 20) at 37°C. The plate was washed as previously, and 100 μL/well of pooled mouse sera (1:100 in 3% BSA/PBS/0.05% Tween 20) were added to the wells and incubated overnight at 4°C. Sera from mice vaccinated with NoV-empty platform and unvaccinated mice (in 3% BSA/PBS/0.05% Tween 20) served as a negative control. Monoclonal anti-M2 antibody (Santa Cruz Biotechnologies, Dallas, USA) served as a positive control. After incubation, the plate was washed as previously, and peroxidase-conjugated AffiniPure goat anti-mouse antibodies (Jackson Immuno Research; in 3% BSA/PBS/0.05% Tween 20) were used for detection. Finally, following the last plate-washing step (6 × 5 min with 200 μL/well), 100 μL/well of HRP substrate solution was added (1-Step Turbo TMB-ELISA, Thermo Scientific), the plate was incubated in the dark until the blue color developed, and the reaction was stopped by adding 50 μL of 0.5 M sulfuric acid to each well. Signal intensity at 450 nm was measured using a plate reader (Epoch, BioTek, Winooski, USA).

### 2.11. Statistical analysis

Collected data were analyzed and visualized using GraphPad Prism (GraphPad Software, United States). Due to the limited number of experimental samples, the normality of the results’ distribution could not be confirmed. Thus, statistical calculations for data obtained in the experiments were performed using two-way RM ANOVA (*p* = 0.05). In the next step, to control the false discovery rate, the two-stage linear step-up procedure of Benjamini, Krieger, and Yekutieli (*p* = 0.05) was carried out. The results from the experiments were compared for all types of sera and additionally between types of virus.

## 3. Results

### 3.1. Design of chimeric calicivirus VLPs presenting influenza virus multivalent M2/NP epitopes

**Figure 1:**
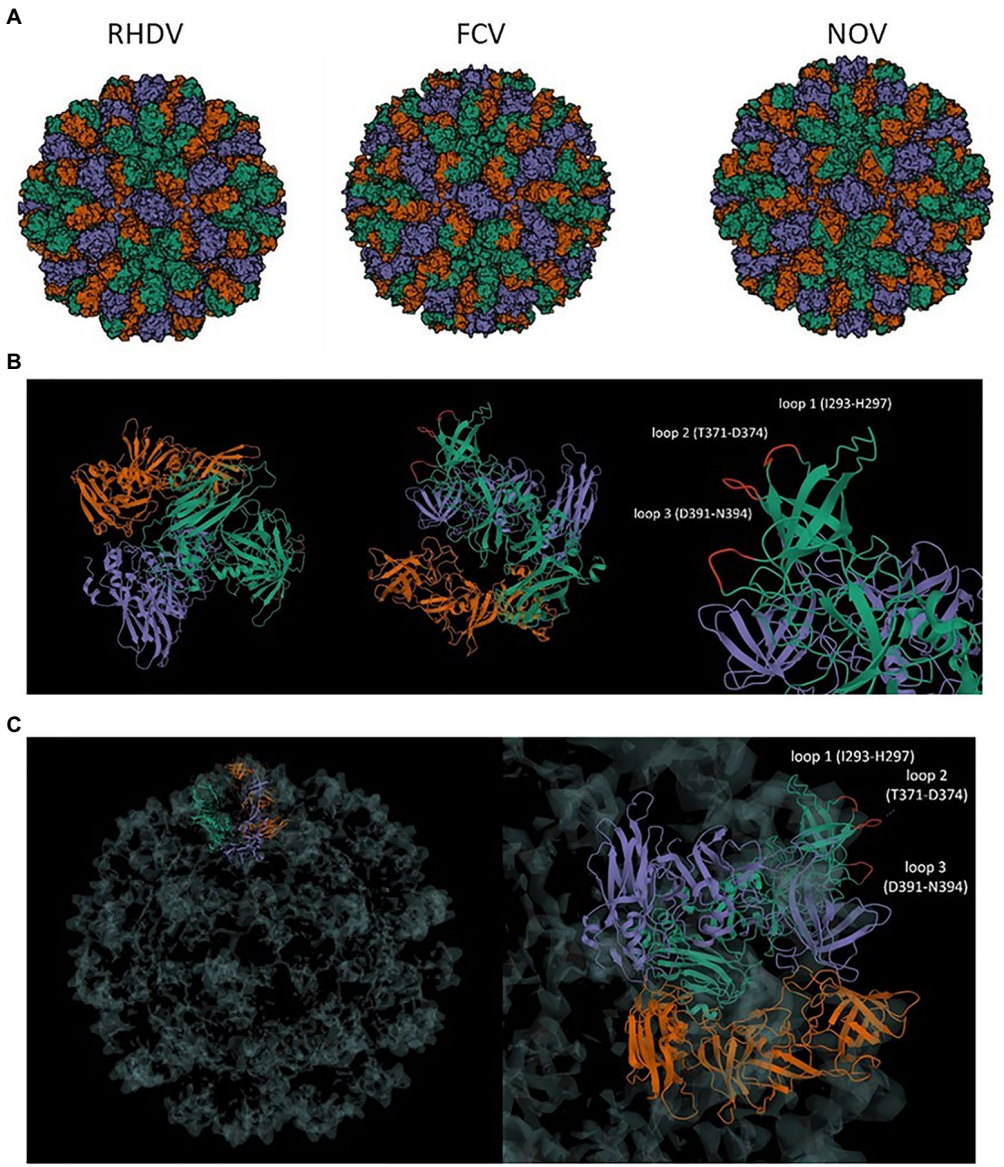
**(A)** Comparison of calicivirus VLP structure. Comparison of VLP of T = 3 symmetry of RHDV (PDB: 3J1P), FCV (PDB: 6GSH), and NoV (PDB: 7MRY); **(B)** VP1 asymmetric unit of NoV T = 3 VLP and localization of the surface loops 1–3; **(C)** Presentation of the VP1 asymmetric unit in T = 3 symmetry structure of VLP and the localization of loops for antigen insertion at the surface of the nanostructure; RHDV, rabbit hemorrhagic disease virus; FCV, feline calicivirus; NoV, norovirus.

The NoV (human calicivirus) major capsid protein, VP1, can self-assemble into VLPs that are structurally similar to native virions. Cryo-electron microscopy of NoV particles revealed three distinct structural loops on the nanostructure’s surface. Multiple copies of those structural loops are presented on the surface of VP1 VLPs due to their structural properties. In this study, the sequence of the *VP1* gene was modified by the insertion of foreign epitopes (antigens) into regions corresponding to those structural loops: loop 1 (I293-H297), loop 2 (T371-D374), and loop 3 (D391-N394; Figure 1). To assess the impact of epitope insertion on the immunogenicity of the cVLPs, the influenza virus multivalent M2/NP epitope was inserted into each of the three structural loops.

**Figure 2:**
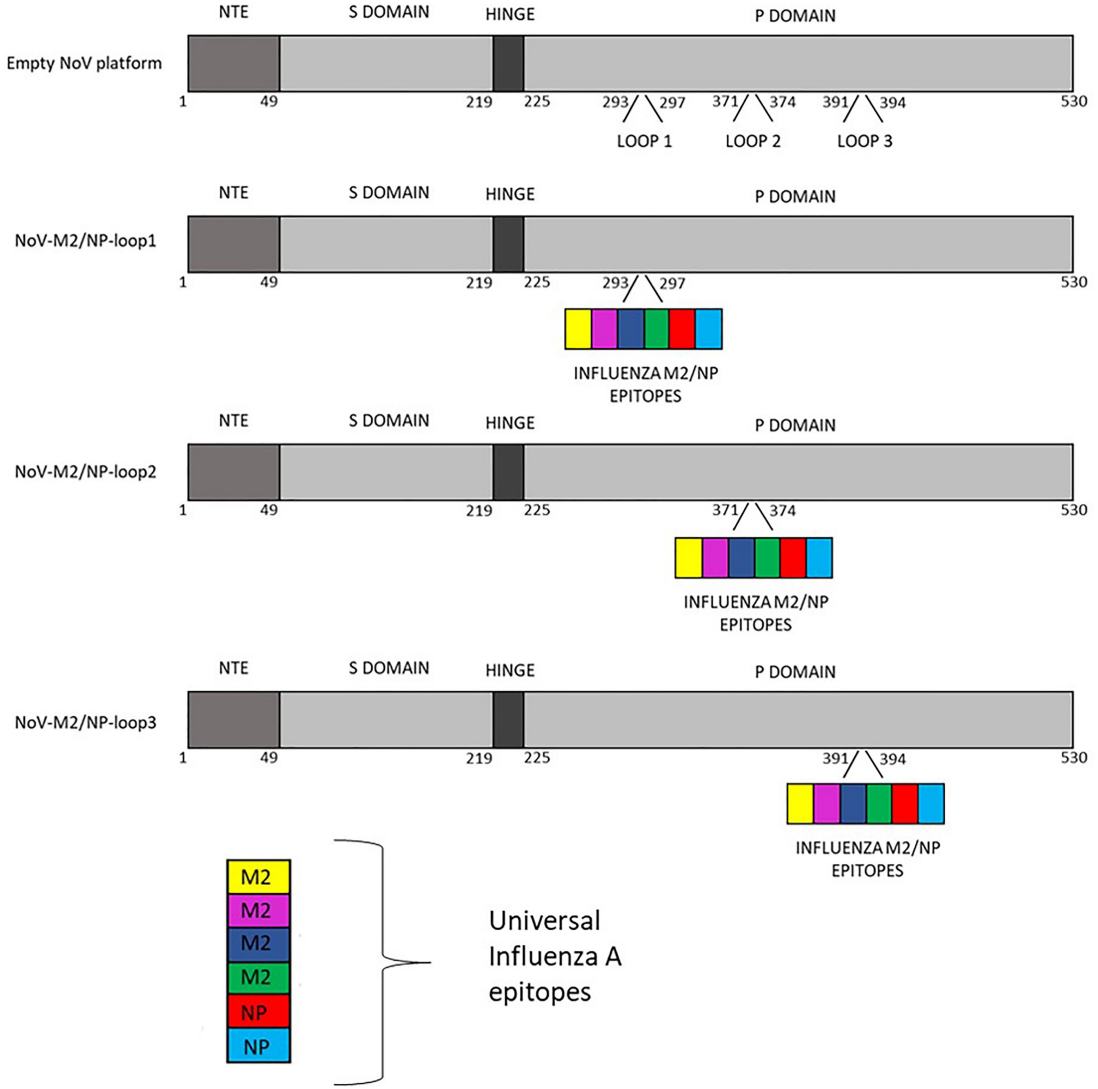
Schematic representation of calicivirus chimeric VLPs presenting influenza A virus multivalent M2/NP epitopes used in this study.

The gene sequence of full-length NoV VP1 capsid protein was designed for epitope insertion. The sequence coding capsid protein was modified by inserting influenza virus multivalent M2/NP epitope in amino acids 293–297 (loop 1), 371–374 (loop 2), or 391–394 (loop 3). Conserved epitopes from M2 and NP proteins are commonly used as conserved protective influenza viral antigens. Due to this strategy for universal vaccine development, tandem repeats of M2 conserved region from influenza strains from different species were chosen. Influenza virus multivalent M2/NP epitope SLLTEVETPIRNE WGCRCNDSSDSLLTEVE TPIRNEWGCRCNG SSDSLLTEVETPTR SEWECRCSDSSDSLL TEVETPTRNEWECRC SDSSDASNENIET MLPFEKSTVM consists of six influenza A virus epitopes from M2 and NP proteins listed in Table 1. A schematic representation of constructs used in this study is presented in Figure 2.

**Table 1:** Amino acid sequences of influenza A virus epitopes.

Strain	Subtype	Origin	Protein	Amino acid sequence
Consensus	H1N1	Human	M2	SLLTEVETPIRNEWGCRCNDSSD
A/Puerto Rico/8/1934	H1N1	Human	M2	SLLTEVETPIRNEWGCRCNGSSD
A/Ontario/309862/2009	H1N1	Swine	M2	SLLTEVETPTRSEWECRCSDSSD
A/Indonesia/560H/2006	H5N1	Avian	M2	SLLTEVETPTRNEWECRCSDSSD
A/chicken/Guiyang/3570/2005	H5N1	Avian	NP	ASNENIETM
A/Resvir-9	H3N2	Human	NP	LPFEKSTVM

**Figure 3:**
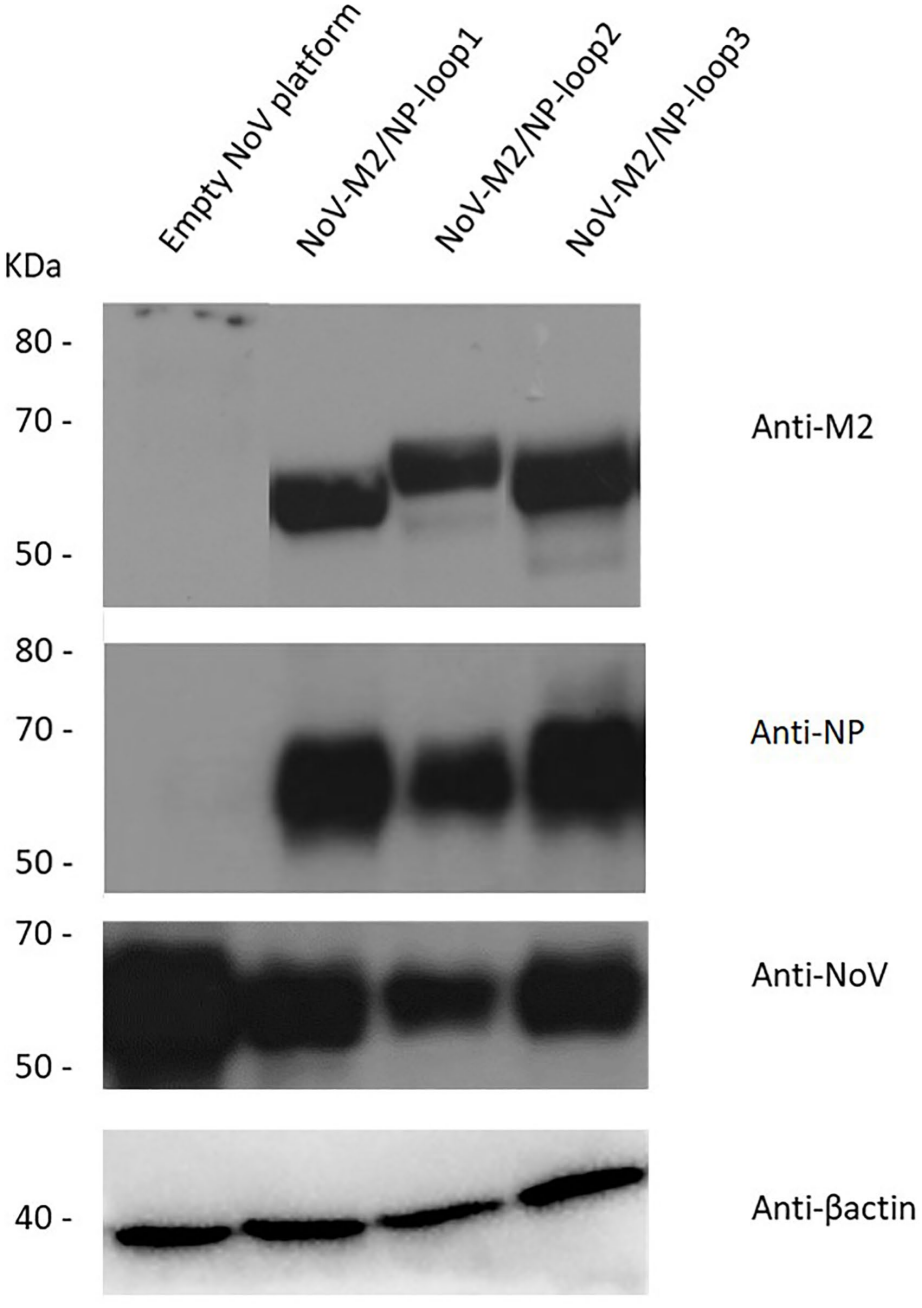
Characterization of the chimeric NoV-M2/NP proteins. Western blotting analysis of different chimeric NoV-M2/NP proteins expressed in the SF9 insect cells in reducing conditions. The protein was detected as a ~ 65 kDa band in culture media using monoclonal anti-M2 antibodies, monoclonal anti-NP antibodies, and anti-VP1-NoV antibodies. β-actin was used as a loading control. Culture media from cells expressing unmodified NoV protein (empty platform) was used as a negative control.

All three modified sequences as well as the unmodified *VP1* gene (empty NoV platform) were optimized using codon adapted for *Spodoptera frugiperda* (*S. Frugiperda*, Sf9) (GeneArt—Thermo Fisher Scientific) and synthesized by Gene Art Gene Synthesis. Synthetic genes were cloned into the multiple cloning site downstream of the polyhedrin promoter of the pFastBac1 expression vector (Invitrogen). Recombinant NoV-M2/NP bacmids were generated using the Bac-to-Bac baculovirus expression system (Invitrogen) and further used to infect Sf9 insect cells. Protein expression was analyzed by Western blotting in reducing conditions. The obtained results confirmed the presence of influenza virus multivalent M2/NP epitope on all three NoV-M2/NP recombinant proteins and not on unmodified NoV VP1 protein (Figure 3).

### 3.2. Purification and characterization of NoV-M2/NP VLPs

**Figure 4:**
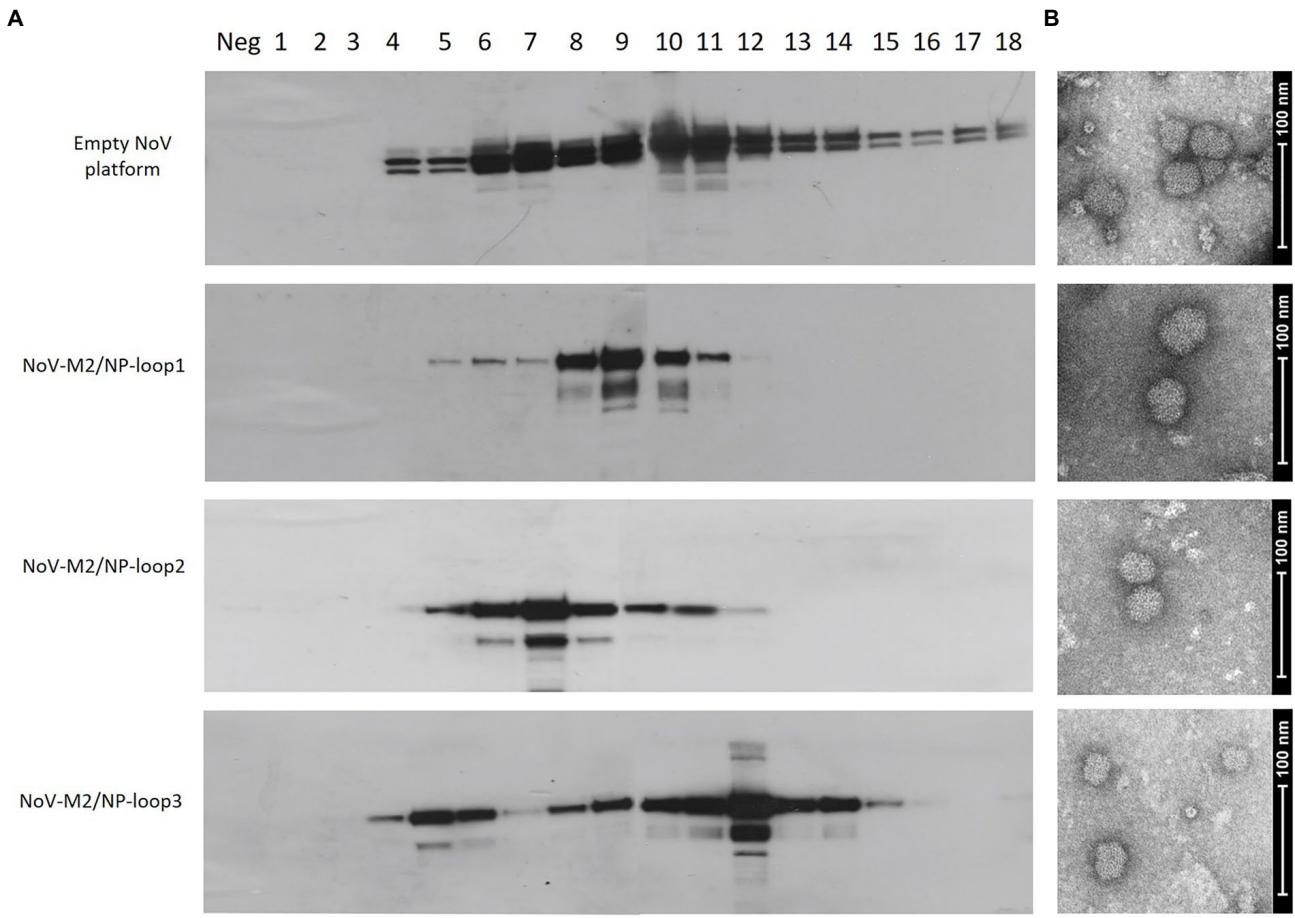
Production and purification of genetically engineered calcivirus chimeric VLPs presenting M2/NP epitopes on their surface. SF9 insect cells were infected with recombinant baculoviruses carrying NoV-M2/NP loop 1–3 constructs. Culture media from the aforementioned cells was harvested and purified using a 26%–50% OptiPrep gradient. **(A)** Western blotting analysis of the sequential fractions collected from ultracentrifugation in OptiPrep gradient. The protein was detected in cell lysates using anti-M2 antibodies. The positive control and reference sample were detected using anti-VP1-NoV antibodies. **(B)** Electron micrograph of purified chimeric NoV-M2/NP loop 1–3 VLPs and empty NoV VLP platform (scale bar: 100 nm).

To obtain a high yield of NoV-M2/NP recombinant proteins, Sf9 insect cell cultures were infected with recombinant baculoviruses at MOI = 5. The 96 h post-infection culture media were harvested and concentrated using Amicon Centrifugal Filter Units (Merck Millipore). To determine whether the NoV-M2/NP recombinant proteins were in the monomer, dimer, or multimeric VLP form, we analyzed the proteins by OptiPrep™ (iodixanol) gradient based on the method described previously (Panasiuk et al., 2022). In brief, Amicon-concentrated samples containing the NoV-M2/NP recombinant proteins were analyzed in a 26%–50% gradient followed by fractionation. Each fraction was then analyzed by SDS-PAGE and Western blotting with an anti-NoV antibody. The obtained results showed clear bands of ~65 kDa proteins within fractions 8–10 for NoV-M2/NP-loop1, 6–9 for NoV-M2/NP-loop2, and 5–6 and 10–14, two separate populations, for NoV-M2/NP-loop3 (Figure 4A). Unmodified NoV VP1 protein served as a positive control and reference sample. To verify the proper assembly and integrity of the produced VLPs, samples containing purified particles were examined using TEM. Images showed spherical particles of about 40 nm, which corresponds to the size of the native NoV platform (Figure 4B).

### 3.3. Mice immunization and analysis of immune response to NoV-M2/NP VLPs

**Figure 5:**
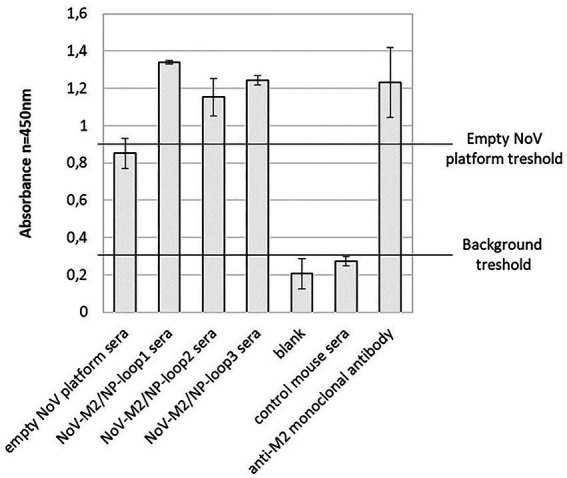
Reactivity of mouse sera collected after immunization with chimeric NoV-M2/NP VLPs by ELISA. An ELISA plate was coated with M2 antigen-presenting VLPs (NoV VLP with M2/NP epitope in all three loops). Pooled sera from immunized mice showed good reactivity with the M2 antigen, as well as to the NoV platform. The bars represent the mean values obtained from triplicate experiments. The mean A450 values and standard deviations are shown on the y-axis.

To compare the immunogenicity of different NoV-M2/NP VLPs produced in insect cells, five groups of BALB/c mice were immunized subcutaneously on days 0, 14, and 28 with purified NoV-M2/NP VLPs. The mice immunized with an empty NoV platform or PBS served as the controls. All mice were immunized in the presence of a squalene-based oil-in-water nanoemulsion adjuvant (AddaVax, InvivoGen). Two weeks after the last vaccination, the blood was collected, and obtained sera were pooled in each group for further analysis. The humoral response induced by immunization was quantified by ELISA assay on a plate coated with M2 antigen-presenting VLPs (NoV VLP with M2/NP epitope in all three loops). Monoclonal anti-M2 antibody was used as a positive control. The results showed that vaccination with all NoV-M2/NP constructs results in a significant anti-M2 response and an anti-NoV response in immunized animals. The reaction observed for VLP-immunized mice was comparable to or higher than the reaction observed for monoclonal anti-M2 antibodies that served as a positive control. There were no significant differences in anti-M2 response between NoV-M2/NP vaccinated mice (Figure 5).

**Figure 6:**
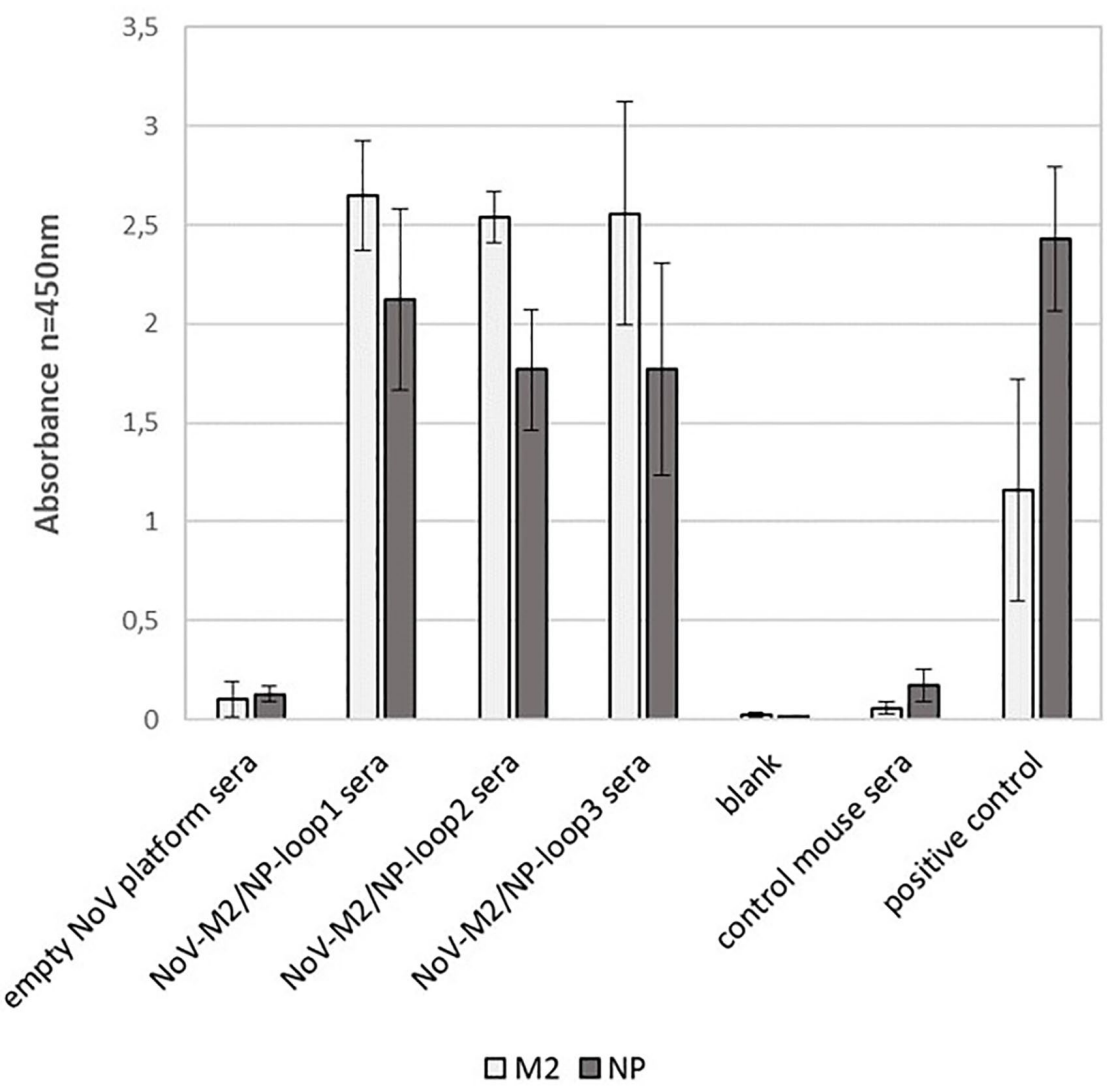
Analysis of the terminal antibody titers in the pooled mouse sera collected after immunization with chimeric NoV-M2/NP VLPs. ELISA plates were coated with M2 antigen-presenting VLPs (NoV VLP with M2/NP epitope in all three loops). The dilution factor of the pooled sera is shown on the x-axis. For each ELISA, the mean value from three independent experiments performed is presented. The mean A450 values and standard deviations are shown on the y-axis.

The end-point serum titrations showed that the NoV-M2/NP antibody titer reached 10^3^ (Figure 6). The antibody titer was estimated as the serum concentration at which the binding was at least two times higher than that of the PBS-adjuvant immunized control serum.

**Figure 7:**
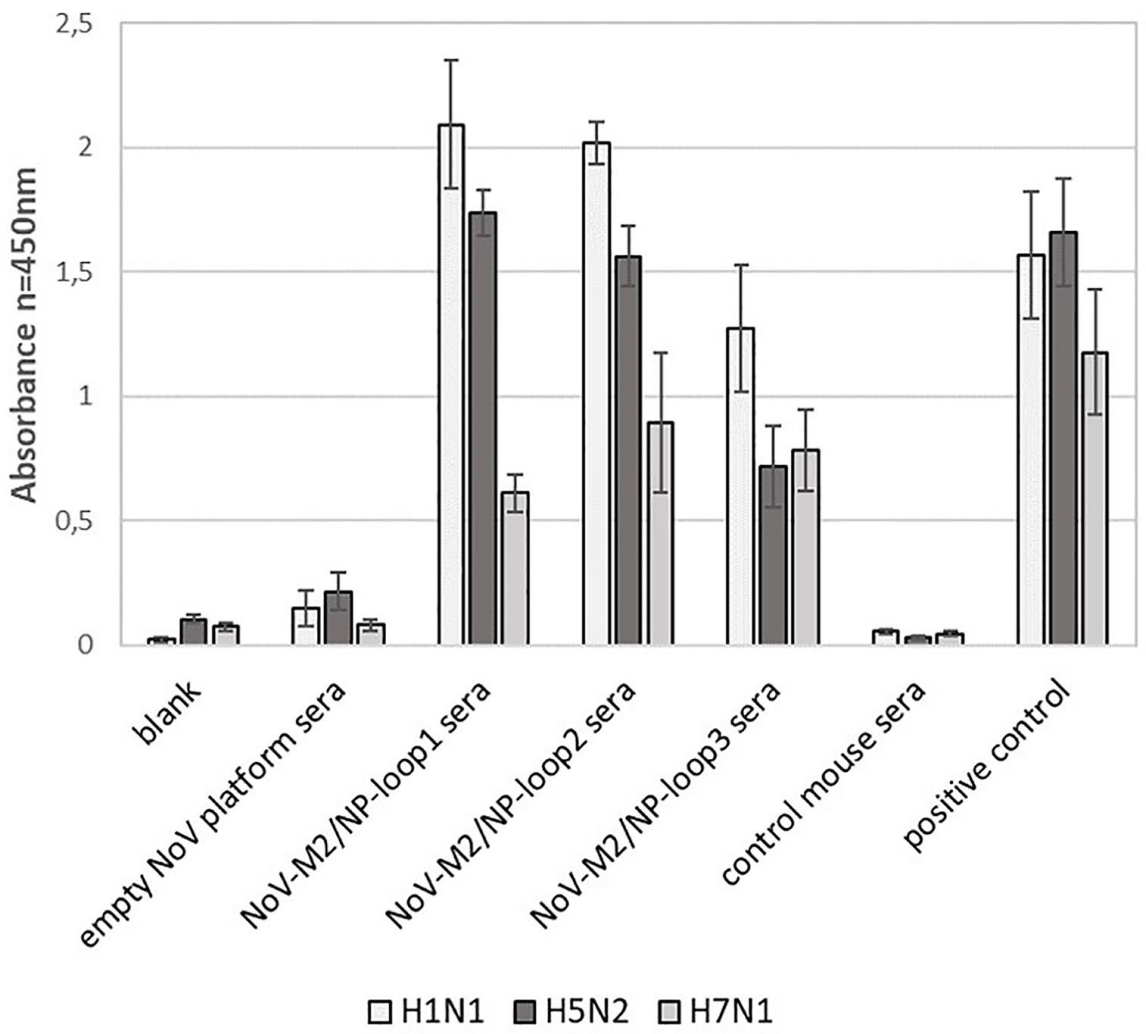
Impact of loop localization on epitope presentation *via* NoV-M2/NP VLPs estimated by peptide ELISA assay. ELISA plates were coated with M2 and NP peptides. For each ELISA, the mean value from three independent experiments performed is presented. The mean A450 values and standard deviations are shown on the y-axis.

To evaluate whether the localization of the M2/NP epitope in different loops on the calicivirus nanoparticle had an impact on epitope presentation, we performed peptide ELISA using ELISA plates coated with synthetic M2 and NP peptides. The analysis showed that all NoV-M2/NP-loop1-3 antibodies had strong reactivity with linear M2 and NP epitopes and no significant differences were detected between the constructs (Figure 7).

**Figure 8:**
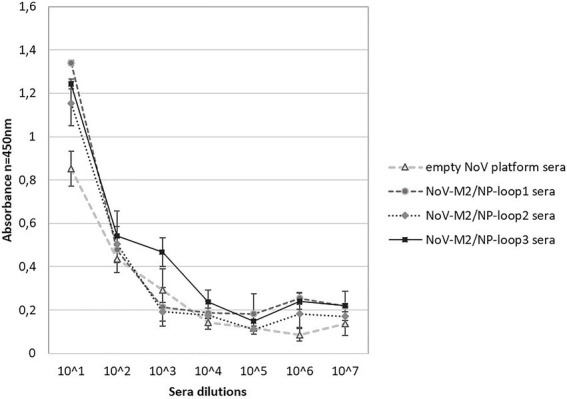
Characterization of immune response after immunization with NoV-M2/NP VLPs toward universal influenza epitopes recognition. ELISA plates were coated with an inactivated virus for influenza A virus of H1N1, H5N2, and H7N1 strains. For each ELISA, the mean value from three independent experiments performed is presented. The mean A450 values and standard deviations are shown on the y-axis.

In addition, obtained mouse sera were analyzed for their ability to recognize universal influenza epitopes from different strains of the influenza A virus. For this, ELISA plates were coated with inactivated influenza A virus lysates of H1N1, H5N2, and H7N1 strains. The analysis showed that all NoV-M2/NP-loop1-3 sera can react specifically with each tested influenza strain suggesting that obtained antibodies carry universal epitope recognition patterns. Moreover, the antibodies obtained by immunization with NoV-M2/NP-loop1 and NoV-M2/NP-loop2 construct showed significantly higher reactivity for H1N1 and H5N2 viruses than NoV-M2/NP-loop3 sera. This suggests that epitopes presented in loop 1 and loop 2 have better conformational presentation allowing for the production of more reactive conformational antibodies (Figure 8).

Data obtained in ELISA tests for recognition of universal influenza epitopes from different strains of influenza A virus were also used in statistical analysis. The first stage of two-way RM ANOVA has shown statistical differences between types of sera (*p* < 0.0001) and types of virus (*p* = 0.0063). Serum (76.84%), viruses (7.97%), and serum–virus interaction (10.97%) were the main causes of data variation which can be seen in ELISA test results (Supplementary File 1). In the next step, a more detailed comparison of mouse sera interaction with the H1N1 virus revealed a lack of statistically significant differences (*p* > 0.05) between the results obtained in the ELISA test for empty NoV platform sera and control mouse sera, NoV-M2/NP-loop1 sera and NoV-M2/NP-loop2 sera, NoV-M2/NP-loop1 sera and positive control, as well as NoV-M2/NP-loop3 sera and positive control. Comparison of H5N2 and sera interaction contained only two records that were not marked as statistically significantly different. Moreover, this discovery appeared similar to the previously described group for NoV-M2/NP-loop1 sera, positive control, and NoV-M2/NP-loop2 sera, positive control comparisons. Statistical analysis for data in group H7N1–sera interaction allowed us to discover only three records that were not statistically different: comparison blank vs. empty NoV platform sera, NoV-M2/NP-loop1 sera vs. NoV-M2/NP-loop2 sera, and NoV-M2/NP-loop2 sera vs. NoV-M2/NP-loop3 sera (Supplementary File 2).

Each kind of sera was employed as a grouping factor for data in the following stage of analysis (Supplementary File 3), and any statistically significant variation in serum interaction with different virus types was searched. Statistical analysis between the results obtained in the ELISA test for each group of sera allowed us to discover differences (*p* < 0.05) for all data in groups blank and NoV-M2/NP-loop2 sera. A lack of statistically significant differences was revealed in entire groups of empty NoV platform sera, NoV-M2/NP-loop3 sera, control mouse sera, and positive control. Mixed results were found in NoV-M2/NP-loop1 sera group where the result of the comparison of H1N1 and H5N2 was not statistically significant and two other comparisons with H7N1 showed strong differences (Supplementary File 4). The full results of the statistical analysis can be found in Supplementary Files.

## 4. Discussion

Calicivirus particles share a similar, highly organized structure that consists of 180 copies of the VP1 major capsid protein with the addition of a small fraction of a minor capsid protein, VP2 (Prasad et al., 1994; Chen et al., 2006; Conley et al., 2019). Prior studies revealed that VP1 and its orthologues can self-assemble into VLPs that closely mimic the structure of the native virus (Prasad et al., 1999; Katpally et al., 2010; Wang et al., 2013; Hu et al., 2022). Analysis of the amino acid sequences of VP1 from different caliciviruses indicates that despite significant variation in the sequences, the basic structure of each monomer consists of three domains—an N-terminal arm (NTA), a shell (S) composed of an eight-stranded β-sandwich, and a protruding domain (P; Figure 1; Chen et al., 2006; Luque et al., 2012). P domain can be further divided into P1 and P2 subdomains from which P1 is moderately conserved, while the region that forms the P2 subdomain exhibits the most variability with major differences within the loops that connect antiparallel β-strands of the protein. Previous studies have suggested that these loops are the immunodominant regions of caliciviruses and that the hypervariable P2 subdomain of VP1 might have a role in antigenicity and interactions with host-cell receptors (Taube et al., 2010; Shanker et al., 2014; Cubillos-Zapata et al., 2020). In addition, structural loops in the P2 subdomain exhibit great flexibility and can tolerate significant sequence alterations without interfering with the basic polypeptide fold of this subdomain. Reconstructions of the VLP with loop insertions revealed analogous T = 3 capsid organization described for wild-type VLPs (Luque et al., 2012). This indicates that surface insertions were dynamic and flexible, allowing for the insertion of short and long epitopes without disrupting the VLP’s spatial structure. This feature of VP1 has been of great interest as a potential insertion site for the design of cVLPs used as a platform or an adjuvant for the delivery of foreign epitopes.

For NoV, three structural loops of the P domain that are present on the surface of the VLPs have previously been described as an insertion site for foreign epitopes. Despite significant sequence variation, other members of the Caliciviridae family, such as RHDV or FCV, share a similar capsid structure with equivalent surface-exposed loops (Figure 1). Up to this date, no studies investigated the differences between the presentation of inserted epitopes and their spatial conformation between the loops (Cubillos-Zapata et al., 2020). In this study, based on the sequence of full-length NoV VP1, we bioengineered three chimeric NoV VLPs that present the influenza multivalent M2/NP epitope in each of the structural loops of the P2 domain of VP1 (Figure 2). All three NoV-M2/NP constructs were successfully produced in insect cells, which are generally considered an efficient and cost-effective expression system. The Western blotting analysis confirmed the correct incorporation of a long (110 amino acids) multivalent influenza virus epitope consisting of tandem repeats of M2/NP epitopes into each of the examined loops. Each of the three chimeric NoV-M2/NP proteins was specifically recognized by anti-M2 and anti-NP antibodies, while NoV VLP (empty platform), which served as a control, was not (Figure 3). Moreover, TEM analysis of proteins purified on OptiPrep gradient confirmed that foreign epitopes were successfully incorporated into the nanoparticles without disrupting the VLP formation and its conformation. Each of the NoV-M2/NP constructs results in the spontaneous formation of spherical VLPs of approximately 40 nm in diameter (Figure 4).

To compare the abilities of each of the NoV-M2/NP-loop1-3 VLPs to stimulate the immune system, BALB/c mice were immunized. In this study, we demonstrated that mice immunized with purified chimeric NoV-M2/NP constructs produce high levels of antibodies specific toward administered antigens (Figures 5, 6). Influenza matrix protein 2 (M2) and nucleoprotein (NP) are considered conserved epitopes found in the vast majority of influenza types and subtypes. M2 epitope has been shown to stimulate general humoral immune responses. In contrast, NP has been shown to strongly stimulate anti-influenza T-cell responses (Tan et al., 2021). Furthermore, NP has a synergistic effect and was previously used as an adjuvant for M2 to boost its immunogenicity in vaccine development strategies. As demonstrated by Mytle and colleagues, combinations of NP and M2 generate serum antibody responses and protect mice against pandemic H1N1 or H5N1 viruses (Mytle et al., 2021). Peggy and colleagues demonstrated that a vaccine containing NP and M2 plasmid DNA protects mice from death and provides some benefits to ferrets (Lalor et al., 2008). The combination of M2 and NP proteins is a promising formulation for a universal influenza vaccine capable of providing broad and long-lasting protection against various influenza viruses (Tutykhina et al., 2018).

cVLPs presenting foreign epitopes have been an object of interest in recent years due to their immunostimulatory properties. VLPs represent PAMPs that are recognized by pattern recognition receptors (PRRs) such as toll-like receptors (TLRs) and are shown to have the ability to initiate the activation of the immune system. Due to those features, VLPs can serve as a platform that presents epitopes and enhances their immunity potential.

Our results show that the immune response induced by chimeric NoV VLPs was targeted toward not only antigens presented in the exposed surface loops but also the platform (NoV VP1 backbone) itself (Panasiuk et al., 2022). These findings suggest that VLPs successfully stimulated the immune system, acting as natural PAMPs and boosting the immune response to the presented foreign epitope.

Epitopes presented in loops 1–3 were easily accessible to effector immune cells, indicating that all of the loops chosen for this study are suitable insertion sites for the induction of specific immune responses against foreign epitopes.

Further analysis revealed that while loop localization did not affect the recognition of synthetic M2 and NP peptides representing linear epitopes (Figure 7), it had a significant impact on the recognition of epitopes present on virus particles (conformational epitopes). In comparison with NoV-M2/NP-loop3 antibodies, antibodies obtained by immunization with NoV-M2/NP-loop1 and NoV-M2/NP-loop2 constructs showed significantly improved reactivity with epitopes present on viral particles from different strains of influenza. This suggests that the epitope inserted in loops 1 and 2 may possess a better conformational presentation and can be used to generate conformational antibodies (Figure 8).

In conclusion, our findings show that chimeric NoV VLPs can be used as a natural immunostimulant and delivery platform for foreign epitopes. All three structural loops in the P2 domain of VP1 are accessible for short and long insertions that do not disrupt VLP assembly and conformation. While all of the studied loops produced high antibody titers after immunization, only loops 1 and 2 were successful in the production of antibodies highly reactive with the conformational epitopes. Furthermore, statistical analysis of the obtained data using two-way RM ANOVA (*p* = 0.05) suggests that loop 1 is supreme in antigen presentation and immunodominance of the obtained immune response. The findings of this study can be used to improve the use of chimeric calicivirus VLPs in vaccine design, epitope delivery, and immune system stimulation in the future.

Conclusively, this study demonstrated the significant potential of calicivirus VLPs as a delivery platform for foreign epitopes and brought new insights into the conformational biology of chimeric VLPs. The findings of this study can further benefit the pursuit of improved vaccine development.

## Data availability statement

The original contributions presented in the study are included in the article/Supplementary material, further inquiries can be directed to the corresponding author.

## Ethics statement

The animal study was reviewed and approved by Committee on the Ethics of Animal Experiments of the Medical University of Gdańsk (Permit Number: 45/2015).

## Author contributions

MP and BG: conceptualization. MP, MC, KZ, LH, VK, GP-S, JW, and BG: methodology. MC: statistical analysis. MP, MC, LH, MN, AK, and BG: visualization. MP, MC, and BG: formal analysis. MP and BG: investigation resources, supervision, project administration, funding acquisition, and writing—original draft preparation. MP, MC, LH, and BG: writing, reviewing, and editing. All authors contributed to the article and approved the submitted version.

## Funding

This work was supported by the Polish National Centre for Research and Development (LIDER/157/L-6/14/NCBR/2015 and POIR.01.01.01-00-1964/20-00).

## Conflict of interest

Authors MP, BG and MC were employed by the company NanoExpo sp. z o.o.

The remaining authors declare that the research was conducted in the absence of any commercial or financial relationships that could be construed as a potential conflict of interest.

## Publisher’s note

All claims expressed in this article are solely those of the authors and do not necessarily represent those of their affiliated organizations, or those of the publisher, the editors and the reviewers. Any product that may be evaluated in this article, or claim that may be made by its manufacturer, is not guaranteed or endorsed by the publisher.
